# Supplementary material for: Dlg Is Required for Short-Term Memory and Interacts with NMDAR in the Drosophila Brain
Source: Int J Mol Sci. 2022 Aug 16;23(16):9187. doi: 10.3390/ijms23169187 (PMC9409279; doi:10.3390/ijms23169187)
Supplement: Supplementary file 1 [file ijms-23-09187-s001.zip › ijms-1794853-supplementary.pdf]

Supplementary figures

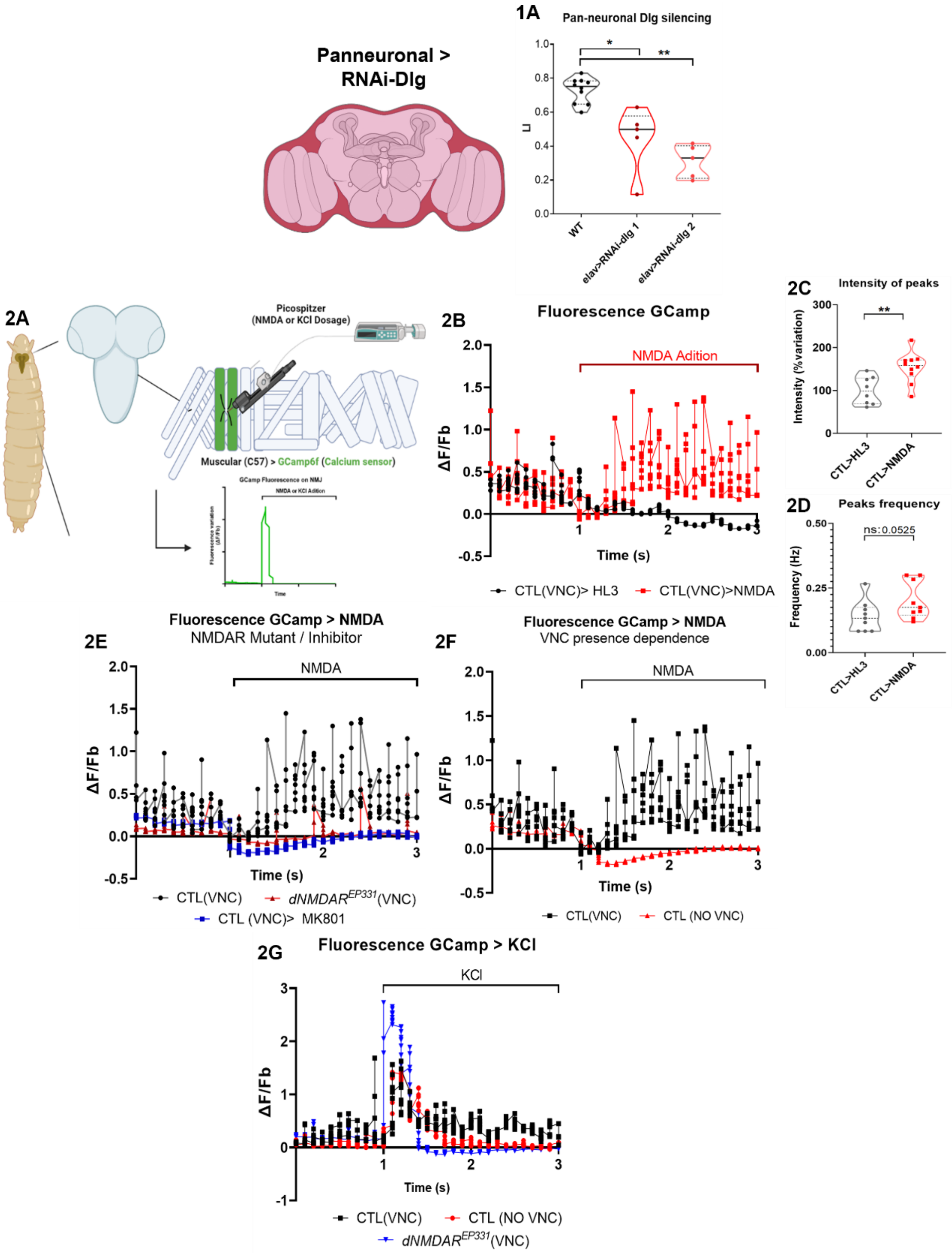

Figure S1. Expression of dsRNA for Dlg decreases de learning index in a short term memory olfactory avoidance test. A) Pan-neuronal expression of dsRNA-*dlg*: two different dsRNA were tested (RNAi-*dlg*1) and RNAi-*dlg*2. All downregulations in the memory experiments were conducted using RNAi-*dlg*2. Brain scheme Created with BioRender.com.

Figure S2. Muscle calcium signals induced by motor neuron's activity are not modulated by NMDA type glutamate receptors. A) Scheme of the larval neuromuscular junction preparation Created with BioRender.com. B) Normalized fluorescence of Gcam6 expressed in muscle, records shown were recorded in muscle with the brain intact. In black or red lines the average recordings were plotted stimulated by a HL3 control solution without Magnesium (black line) or by NMDA (red line) 1 mM in the same solution. NMDA induces a strong response of increased amplitude and frequency of calcium signals. C) quantification of the peak of the GCamp fluorescence in control solution or in NMDA. D) quantification of the frequency of the GCamp fluorescence in control solution or in NMDA. E) Normalized fluorescence of Gcamp6 expressed in muscle, records shown were recorded in muscle with the brain intact. In black or red lines the average recordings were plotted stimulated by a HL3 control solution without Magnesium (black line) in a WT strain or by NMDA 1 mM in the same solution in a NMDAR hypomorphic mutant (red line). The control strain was also tested stimulated by NMDA 1 mM (as in A) but in the presence of the NMDAR inhibitor MK801 (blue line). F) Normalized fluorescence of Gcamp6 expressed in muscle, records shown were recorded in muscle with the brain intact (black line) or in larvae without the brain (red line) stimulated by HL3 control without Magnesium and NMDA 1 mM. F) Normalized fluorescence of Gcamp6 expressed in muscle, records shown were recorded in muscle with the brain intact (red line) or with brain removed (black lines) in control larvae or hypomorphic mutants for NMDARs (green lines) stimulated by 100 mM KCl in HL3 solution without Magnesium (black line). NMDA induces a strong response of increased amplitude and frequency of calcium signals in control larvae recorded with the brain. NMDA is unable to induce calcium signals in the muscle in hypomorphic strain larvae, with an inhibitor of the NMDAR or notable in the absence of the brain. All preparations responded to a KCl stimulus. .
